# Isolation, Molecular Characterization, and Antimicrobial Resistance of Selected Culturable Bacteria From Crayfish (*Procambarus clarkii*)

**DOI:** 10.3389/fmicb.2022.911777

**Published:** 2022-06-07

**Authors:** Zixian Huang, Yuanyuan Li, Chang Cai, Ning Dong

**Affiliations:** ^1^College of Agriculture and Life Sciences, Cornell University, Ithaca, NY, United States; ^2^Department of Medical Microbiology, Experimental Center, Medical College of Soochow University, Suzhou, China; ^3^China-Australian Joint Laboratory for Animal Health Big Data Analytics, College of Animal Science and Technology & College of Veterinary Medicine, Zhejiang A&F University, Hangzhou, China; ^4^Department of Medical Microbiology, School of Biology and Basic Medical Science, Medical College of Soochow University, Suzhou, China

**Keywords:** red swamp crayfish, *Procambarus clarkii*, culturable bacteria, diversity, antimicrobial resistance, whole-genome sequencing

## Abstract

Red swamp crayfish (*Procambarus clarkii*) have become one of the favorite aquatic products in China. The modern farming mode which uses antibiotics to prevent diseases could impact the bacteria in crayfish intestines. Here, we determined the distribution and antimicrobial resistance phenotypes of the selected culturable bacteria in crayfish intestines and characterized an isolate with last-line antibiotic resistance determinant. Totally, 257 strains were isolated from 115 crayfish. These strains were highly diverse, with *Citrobacter* sp. (*n* = 94, 36.6%) and *Aeromonas* sp. (*n* = 88, 34.2%) being dominant. Other isolates belonged to genera *Pseudomonas*, *Myroides*, *Morganella*, *Klebsiella*, *Acinetobacter*, *Proteus*, *Enterobacter*, *Kluyvera,* and *Escherichia*. Most strains from crayfish were susceptible to all tested antibiotics. None of the isolates carried last-line antibiotic resistance genes except one *Escherichia coli* isolate with *bla*_NDM-5_ was detected, which is the first report of *bla*_NDM-5_-positive *E. coli* isolate from red swamp crayfish. Whole-genome sequencing suggested it belonged to ST48 and carried several resistance genes. *bla*_NDM-5_ was located within an Tn*3000*-like transposon linked to an external 5 bp sequence (ACTAT) on both sides on a IncHI1B/HI1A/FIA multi-replicon plasmid. This transposon was considered to be acquired by replicative transposition mediated by IS*3000*. The emergence of bacteria with last-line antibiotic resistance genes in crayfish poses serious threat to public health since crayfish could act as a reservoir for the transfer of resistance to humans.

## Introduction

Red swamp crayfish (*Procambarus clarkii*) are freshwater crustaceans invading shallow lakes, streams, ditches, and estuaries. Originally distributed in North America and introduced into China from Japan since the late 1930s, red swamp crayfish have been commercially farmed and become one of the favorite aquatic products in China (Wang et al., 2018; Jin et al., 2019). In 2020, crayfish has been extensively farmed in 23 provinces with a total aquaculture production of 2.39 million tons in China. The top five crayfish-breeding provinces in China are Hubei, Anhui, Jiangsu, Hunan, and Jiangxi, which accounted for 94% of the output in total (Ministry-of-Agriculture-and-Rural-Affairs-of-the-People’s-Republic-of-China, 2021). Rice-crayfish mixed farming which constitutes a highly efficient artificial ecosystem has become the dominant cultivation method nowadays (Tan et al., 2021). This integrated cultivation mode routinely uses antibiotics such as norfloxacin, nitrofurazone to prevent and treat diseases in aquaculture systems during the breeding process (Sun et al., 2019). The application of antibiotics could lead to the emergence of antimicrobial resistance even at very low drug concentrations in aquatic environments, posing a severe threat to humans, other animals, and to ecological sustainability (Sun et al., 2020).

The intestinal microflora of red swamp crayfish was reported to be highly diverse, with Proteobacteria, Tenericutes, Firmicutes, and Bacteroidetes being the dominant phyla (Shui et al., 2020; Zhang et al., 2020). Previous studies have isolated diverse bacterial species from the intestine of crayfish, including those belonging to *Acinetobacter*, *Aeromonas*, *Citrobacter*, *Bacillus*, *Corynebacterium*, *Flavobacterium*, *Micrococcus*, *Pseudomonas*, *Staphylococcus,* and *Vibrio* (Lim et al., 2020; Liu et al., 2020; Feng et al., 2021; Wu et al., 2021). Bacteria from freshwater animals such as *Aeromonas* sp., *Citrobacter* sp., and *Vibrio* sp. have been demonstrated to be vectors for antimicrobial resistance genes (Ranjbar et al., 2019; Singh et al., 2020; Sony et al., 2021). Drug-resistant bacteria could transmit from crayfish to human through the food chain, incurring infectious disease among humans. Carbapenems, colistin, and tigecycline are last-resort antibiotics for treatments of infections caused by multiple drug-resistant Gram-negative bacteria (Zhang et al., 2018). However, the emergence of carbapenemase genes, mobile colistin resistance genes (*mcr-1*), and the recently reported tigecycline resistance determinants [*tet*(X), *tmexCD1*-*toprJ1*] significantly comprised the efficacy of these last-line antibiotics (Liu et al., 2016; He et al., 2019; Sun et al., 2019; Lv et al., 2020). The antimicrobial susceptibility and the presence of last-line antibiotic resistance genes of bacteria from crayfish intestines remained rarely investigated. To fill this gap, we isolated bacteria from the intestine of red swamp crayfish, characterized the antimicrobial resistance properties, and detected the presence of last-line antibiotic resistance genes of the selected culturable bacteria, and characterized strain with last-line antibiotic resistance genes using genomics-based approaches. In this study, we first reported a *bla*_NDM-5_-positive *Escherichia coli* isolate from red swamp crayfish. *bla*_NDM-5_ could be acquired by the plasmid in this *E. coli* strain through replicative transposon mediated by IS*3000*. The emergence of multidrug resistant bacteria particularly that with last-line antibiotic resistance genes in crayfish intestine pose serious threat on public health since crayfish could act as a reservoir for the transfer of antimicrobial resistant bacteria and antimicrobial resistance genes to humans.

## Materials and Methods

### Sample Collection, Strain Isolation, and Identification

A total of 115 healthy red swamp crayfish each weighing approximately 15–20 g were purchased in July 2020 at one time from aquaculture market in Hangzhou, Zhejiang province, China. The crayfish were all dark red and shiny, with complete appendages, smooth body surface, no ciliates and other attachments, strong activity ability, and sensitive response. The entire body of the crayfish was sterilized with 75% ethanol, and then, the intestines in the crayfish were cut open with sterile scissors and maintained in 2 ml sterilized tubes. After rinsing with 0.85% sterile saline and triturating with inoculation loops, 5 μl mashing liquid was inoculated onto the Salmonella Shigella (SS) agar plate for purification. The rest of the mashing intestinal liquid were transferred and cultivated in broth for enrichment. The crayfish intestine samples were cultivated in incubator with constant temperature of 35°C for 18–20 h. A 5 μl broth samples were inoculated onto SS agar plates for further isolation. All visible single colonies were spread on SS plates for purification. The species of all isolates were identified using the matrix-assisted laser desorption/ionization mass spectrometry (MALDI-TOF MS, Bruker Daltonics, Billerica, MA, United States).

### Antimicrobial Susceptibility Testing

The antimicrobial susceptibility of the selected culturable isolates from crayfish intestine against eight commonly used antibiotics including ampicillin, gentamicin, ciprofloxacin, cefotaxime, trimethoprim-sulfamethoxazole, cefoperazone-sulbactam, meropenem, and tetracycline was tested using the Kirby-Bauer disk diffusion method (Hudzicki, 2009). The observed inhibition zone diameters were recorded and interpreted according to the CLSI guidelines (CLSI, 2020). Disk diffusion testing for *Myroides* sp. isolates was not performed since the disk diffusion method has not been systematically studied on them. The minimum inhibitory concentration (MICs) of 15 commonly antibiotics (imipenem, meropenem, ertapenem, cefmetazole, ceftazidime, cefotaxime, amikacin, aztreonam, piperacillin-tazobactam, cefoperazone-sulbactam, ceftazidime-avibactam, cefepime, colistin, tigecycline, and ciprofloxacin) against the *bla*_NDM-5_-positive *E. coli* isolate was tested using microbroth dilution method with *E. coli* ATCC 25922 as control according to the CLSI guidelines (CLSI, 2020).

### Screening of Last-Line Antibiotic Resistance Genes

Genes encoding last-line antibiotic resistance were screened by PCR and Sanger sequencing using primers described previously (Supplementary Table 1), including carbapenemase genes (*bla*_VIM_, *bla*_NDM_, *bla*_IMP_, *bla*_OXA_, and *bla*_KPC_), mobile colistin resistance genes (*mcr*), plasmid-mediated high-level tigecycline resistance genes (*tet*(X)), and the recently reported plasmid-encoded resistance-nodulation-division efflux pump gene *tmexCD1*-*toprJ1* conferring tigecycline resistance (Poirel et al., 2011; Liu et al., 2016; He et al., 2019; Sun et al., 2019; Lv et al., 2020; Li et al., 2021). Strain(s) with last-line antibiotic resistance gene(s) was subjected to further characterization.

### Whole-Genome Sequencing and Bioinformatics Analysis

Genomic DNA was extracted from the *E. coli* isolate carrying *bla*_NDM-5_ using the Magen DNA extraction kit (Magen, Guangzhou, China) according to the manufacturer’s instructions. Next-generation sequencing was performed using both the Illumina Novoseq 6,000 platform and the Oxford nanopore Technologies MinION platform using method described previously (Li et al., 2018). Hybrid genome sequence assembly with reads from both platforms was conducted using Unicycler v 0.4.4 (Wick et al., 2017). Assembled genome sequence was annotated with the RAST tool and modified manually (Aziz et al., 2008). Multi-locus sequence typing was conducted using the MLST tool v2.16 (Seemann, 2021). Antimicrobial resistance gene was identified using ResFinder 2.0 (Kleinheinz et al., 2014). Plasmid replicons were analyzed using PlasmidFinder v2.1 (Carattoli et al., 2014). Insertion sequences were identified using ISfinder (Siguier et al., 2006). Plasmid map and genetic context comparisons were visualized using DNAPlotter and Easyfig, respectively (Carver et al., 2009; Sullivan et al., 2011).

### Nucleotide Sequence Accession Numbers

The complete genome sequence of *E. coli* strain X14-3 was deposited in GenBank with accession numbers CP084055 (chromosome), CP084057 (plasmid pX14-3-NDM), and CP084056 (plasmid pX14-3-tetA) under BioProject accession PRJNA765572.

## Results

### Diversity of the Selected Culturable Bacteria From Crayfish Intestine

The species and number of bacteria isolated from red swamp crayfish are shown in Table 1. A total of 257 strains were isolated from 115 crayfish, including 121 *Enterobacteriaceae* and 136 other bacteria. One to six strains of bacteria were isolated from 35, 46, 19, 8, 1, and 6 of crayfishes, respectively (Supplementary Table 2). These strains were highly diverse. *Citrobacter* sp., with a total number of 94, accounted for most of the isolated strains. These *Citrobacter* sp. strains were isolated from 83 crayfish and belonged to *C. freundii* (*n* = 60), *C. braakii* (*n* = 32), and *C. youngae* (*n* = 2). Other species isolated included *Aeromonas* sp., *Pseudomonas* sp., *Myroides odoratimimus*, *Morganella morganii*, *Klebsiella* sp., *Acinetobacter* sp., *Proteus* sp., *Enterobacter* sp., *Kluyvera* sp., and *E. coli*. These *Aeromonas* sp. strains were belonged to *A. veronii* (*n* = 40), *A. caviae* (*n* = 16), *A. enteropelogenes* (*n* = 15), *A. hydrophila* (*n* = 11), and *A. jandaei* (*n* = 6). *Pseudomonas* sp. strains included *P. putida* (*n* = 17), *P. monteilii* (*n* = 3), *P. mosselii* (*n* = 2), *P. otitidis* (*n* = 1), and other *Pseudomonas* sp. (*n* = 6). *Klebsiella* sp. were belonged to *K. pneumoniae* (*n* = 7) and *K. aerogenes* (*n* = 1). *Acinetobacter* sp. were belonged to *A. radioresistens* (*n* = 1), *A. lwoffii* (*n* = 1), *A. baumannii* (*n* = 1), and other *Acinetobacter* sp. (*n* = 3). *Proteus* sp. strains were belonged to *P. vulgaris* (*n* = 2), *P. hauseri* (*n* = 1), and other *Proteus* sp. (*n* = 1). *Kluyvera* sp. included *K. crycrescens* (*n* = 1) and *K. ascorbate* (*n* = 1).

**Table 1:** Species and number of bacteria isolated from red swamp crayfish^*^.

Family	Species	Number of isolates	Number of crayfish	Isolation rate (%)
Enterobacteriaceae	*Citrobacter braakii*	32	32	27.8%
	*Citrobacter freundii*	60	60	52.2%
	*Citrobacter youngae*	2	2	1.7%
	*Morganella morganii*	10	10	8.7%
	*Klebsiella pneumoniae*	7	7	6.1%
	*Klebsiella aerogenes*	1	1	0.9%
	*Proteus vulgaris*	2	2	1.7%
	*Proteus hauseri*	1	1	0.9%
	*Proteus mirabilis*	1	1	0.9%
	*Kluyvera cryocrescens*	1	1	0.9%
	*Kluyvera georgiana*	1	1	0.9%
	*Escherichia coli*	3	3	2.6%
Non-Enterobacteriaceae	*Aeromonas veronii*	40	40	34.8%
	*Aeromonas enteropelogenes*	15	15	13.0%
	*Aeromonas hydrophila*	11	11	9.6%
	*Aeromonas caviae*	16	16	13.9%
	*Aeromonas jandaei*	6	6	5.2%
	*Pseudomonas putida*	17	17	14.8%
	*Pseudomonas monteilii*	3	3	2.6%
	*Pseudomonas mosselii*	2	2	1.7%
	*Pseudomonas otitidis*	1	1	0.9%
	Other *Pseudomonas* sp.	6	6	5.2%
	*Myroides odoratimimus*	13	13	11.3%
	*Acinetobacter radioresistens*	1	1	0.9%
	*Acinetobacter lwoffii*	1	1	0.9%
	*Acinetobacter baumannii*	1	1	0.9%
	Other *Acinetobacter* sp.	3	3	2.6%

* Number of crayfish indicates the number of crayfish that the corresponding bacterial species were isolated from. Isolation rate was calculated using the number of crayfish from which a specific bacterial species was isolated divided by the number of all crayfish (*n* = 115).

### Antimicrobial Resistance Profiles of Bacteria From Crayfish Intestine

The antimicrobial resistance profiles of the selected culturable bacteria from red swamp crayfish are shown in Table 2. All Enterobacteriaceae strains were susceptible or intermediate to gentamicin, trimethoprim-sulfamethoxazole, cefoperazone-sulbactam, meropenem, and tetracycline. Enterobacteriaceae strains resistant to ampicillin, ciprofloxacin, and cefotaxime accounted for 29.8, 4.1, and 2.5%, respectively. All Enterobacteriaceae from crayfish intestine were susceptible to all antibiotics tested or resistant to one or two of the antibiotics ampicillin, ciprofloxacin, cefotaxime, and trimethoprim-sulfamethoxazole. Except the 8 *Klebsiella* sp. and 2 *Kluyvera* sp. strains resistant to ampicillin and 1 *E. coli* strain resistant to ampicillin and cefotaxime, all other antibiotic resistant Enterobacteriaceae strains belonged to *Citrobacter* sp.

**Table 2:** Antimicrobial resistance profiles of culturable bacteria from red swamp crayfish.

Antibiotics	Enterobacteriaceae				Non-Enterobacteriaceae^*^			
	Zone diameter (range, mm)	R%	I%	S%	Zone diameter (range, mm)	R%	I%	S%
Ampicillin	6 ~ 24	29.8	53.2	17.0	6 ~ 23	90.0	0.0	10.0
Gentamicin	20 ~ 28	0.0	0.0	100	6 ~ 28	5.0	0.0	95.0
Ciprofloxacin	12 ~ 40	4.1	0.0	95.9	23 ~ 44	0.0	0.0	100.0
Cefotaxime	22 ~ 38	2.5	0.0	97.5	6 ~ 38	10.0	0.0	90.0
Trimethoprim-sulfamethoxazole	12 ~ 30	0.0	4.3	95.7	6 ~ 30	10.0	5.0	85.0
Cefoperazone-sulbactam	22 ~ 36	0.0	0.0	100.0	6 ~ 32	10.0	5.0	85.0
Meropenem	25 ~ 36	0.0	0.0	100.0	19 ~ 36	10.0	30.0	60.0
Tetracycline	20 ~ 32	0.0	0.0	100.0	6 ~ 33	5.0	0	95

* Disk diffusion testing for *Myroides* sp. was not performed since this method has not been systematically studied on this species.

All non-Enterobacteriaceae strains were susceptible to ciprofloxacin, and that resistant to ampicillin, gentamicin, cefotaxime, trimethoprim-sulfamethoxazole, cefoperazone-sulbactam, meropenem, and tetracycline accounted for 90.0, 5.0, 10.0, 10.0, 10.0, 10.0, and 5.0%, respectively. The majority of non-Enterobacteriaceae strains was susceptible to all antibiotics tested, but strains resistant to multiple antibiotics were observed, including a *Pseudomonas* sp. strain resistant to ampicillin, cefotaxime, trimethoprim-sulfamethoxazole, cefoperazone-sulbactam, and meropenem, and an *A. caviae* strain resistant to gentamicin, cefotaxime, trimethoprim-sulfamethoxazole, cefoperazone-sulbactam, and tetracycline. In addition, the antimicrobial resistance profiles of predominate bacterial species from red swamp crayfish are shown in Table 3.

**Table 3:** Antimicrobial resistance profiles of predominate bacterial species from red swamp crayfish.

Antibiotics	*Citrobacter* *freundii*			*Citrobacter* *braakii*			*Aeromonas* *veronii*			*Aeromonas enteropelogenes*			*Aeromonas* *caviae*			*Pseudomonas* *putida*			*Klebsiella pneumoniae*		
	R%	I%	S%	R%	I%	S%	R%	I%	S%	R%	I%	S%	R%	I%	S%	R%	I%	S%	R%	I%	S%
Ampicillin	20.0	32.0	48.0	28.1	43.8	28.1	100.0	0.0	0.0	26.7	0.0	73.3	100.0	0.0	0.0	23.5	0.0	76.5	100.0	0.0	0.0
Gentamicin	0.0	0.0	100.0	0.0	0.0	100.0	0.0	0.0	100.0	0.0	0.0	100.0	31.3	0.0	68.7	0.0	0.0	100.0	0.0	0.0	100.0
Ciprofloxacin	6.7	0.0	93.3	0.0	0.0	100.0	0.0	0.0	100.0	0.0	0.0	100.0	0.0	0.0	100.0	0.0	0.0	100.0	0.0	0.0	100.0
Cefotaxime	3.3	0.0	96.7	0.0	0.0	100.0	0.0	0.0	100.0	0.0	0.0	100.0	18.8	0.0	81.2	17.6	0.0	82.4	0.0	0.0	100.0
Trimethoprim-sulfamethoxazole	0.0	0.0	100.0	0.0	0.0	100.0	0.0	10.0	90.0	26.7	0.0	73.3	25.0	0.0	75.0	5.9	0.0	94.1	0.0	42.9	57.1
Cefoperazone-sulbactam	0.0	0.0	100.0	0.0	0.0	100.0	0.0	0.0	100.0	26.7	0.0	73.3	18.8	0.0	81.2	0.0	23.5	76.5	0.0	0.0	100.0
Meropenem	0.0	0.0	100.0	0.0	0.0	100.0	12.5	32.5	55.0	0.0	0.0	100.0	0.0	18.8	81.2	0.0	0.0	100.0	0.0	0.0	100.0
Tetracycline	0.0	0.0	100.0	0.0	0.0	100.0	0.0	0.0	100.0	0.0	0.0	100.0	18.8	0.0	81.2	0.0	0.0	100.0	0.0	0.0	100.0

### Characteristics of an *Escherichia coli* Isolate Carrying *bla*_NDM-5_

PCR screening indicated one *E. coli* isolate (X14-3) carried *bla*_NDM-5_, and other isolates were negative for last-line antibiotic resistance genes including carbapenemases genes, *mcr-1*, *tet*(X), and *tmexCD1*-*toprJ1*. The crayfish isolated with this *E. coli* strain also has the following isolates: *Citrobacter braakii*, *Aeromonas veronii*, *Myroides odoratimimus*, *Pseudomonas mosselii,* and *Pseudomonas putida* (Supplementary Table 2). Antimicrobial susceptibility test suggested *E. coli* strain X14-3 was susceptible to most antibiotics including imipenem (≤1 μg/ml), meropenem (≤1 μg/ml), ertapenem (≤2 μg/ml), cefmetazole (≤2 μg/ml), piperacillin-tazobactam (16/4 μg/ml), ceftazidime-avibactam (≤0.5/4 μg/ml), cefepime (≤4 μg/ml), colistin (1 μg/ml), tigecycline (≤0.25 μg/ml), ciprofloxacin (≤1 μg/ml), amikacin (≤4 μg/ml), aztreonam (≤4 μg/ml), but was resistant to ceftazidime (>128 μg/ml), and cefotaxime (64 μg/ml). Despite carrying the carbapenemase gene *bla*_NDM-5_, strain X14-3 remained susceptible to carbapenems. The underlying mechanism remained to be investigated.

**Figure 1:**
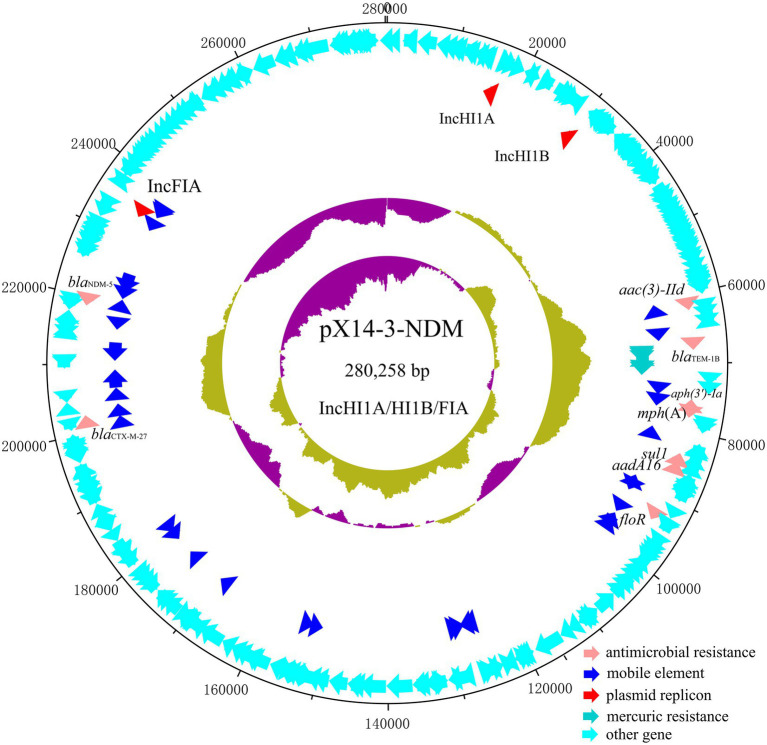
Circular plasmid map of pX14-3-NDM. Pink, blue, red, turquoise, and cyan arrows represent genes responsible for antimicrobial resistance, mobile element, plasmid replicon, mercuric resistance, and other functions, respectively.

**Figure 2:**
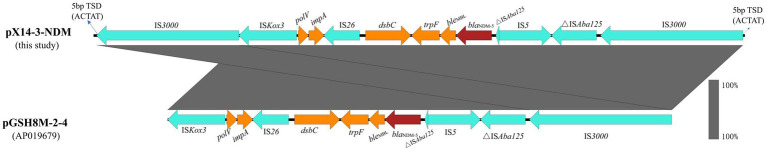
Genetic environment of *bla*_NDM-5_. Red, cyan, and yellow arrows denote antimicrobial resistance genes, mobile elements, and other genes, respectively. TSD, target site duplication.

The genome of strain X14-3 was assembled into three complete circularized contigs, including a 4,566,038 bp chromosome encoding 4,492 predicted ORFs with a GC content of 50.9% and two multidrug resistant plasmids (pX14-3-NDM and pX14-3-tetA). Strain belonged to ST48 and carried an array of resistance genes on both plasmids. Plasmid pX14-3-NDM was 280,258 bp in length with a G + C content of 48.2%. It was an IncHI1A/HI1B/FIA multi-replicon plasmid comprising 315 predicted ORFs. A BLASTn search in the NCBI nr database suggested it was a plasmid which exhibited 99.89% identity with the 411,833 bp plasmid p4M8F (accession: MN256758) from an *E. coli* isolate at 67% coverage. pX14-3-NDM carried antimicrobial resistance genes *bla*_NDM-5_, *bla*_CTX-M-27_, *aac(3)-Ild*, *bla*_TEM-1B_, *aph(3′)-Ia*, *mph*(A), *sul1*, *aadA16,* and *floR* (Figure 1). Besides, pX14-3-NDM carried a mercury resistance (*mer*) operon. The *bla*_NDM-5_ gene was located within a 14,678 bp intact Tn*3000*-like transposon with the structure IS*3000*-IS*Kox3*-*polV*-*impA*-IS*26*-*dsbC*-*trpF*-*ble*_MBL_-*bla*_NDM-5_-△IS*Aba125*-IS*5*-△IS*Aba125*-IS*3000*. This region was flanked by two copies of IS*3000*, one at each end in the same orientation. It was highly homologous to its counterparts on several plasmids including pGSH8M-2-4 (accession: AP019679, 99.98% sequence identity and 100% coverage) in the NCBI nr database except that sequences in the database carried only one copy of IS*3000* located upstream of *bla*_NDM-5_. Each IS*3000* in pX14-3-NDM was in turn linked to an external 5 bp sequence (ACTAT), possibly a product of target site duplications, suggesting that the Tn*3000*-like transposon was inserted into the backbone of plasmid pX14-3-NDM by replicative transposition mediated by IS*3000* (Figure 2).

**Figure 3:**
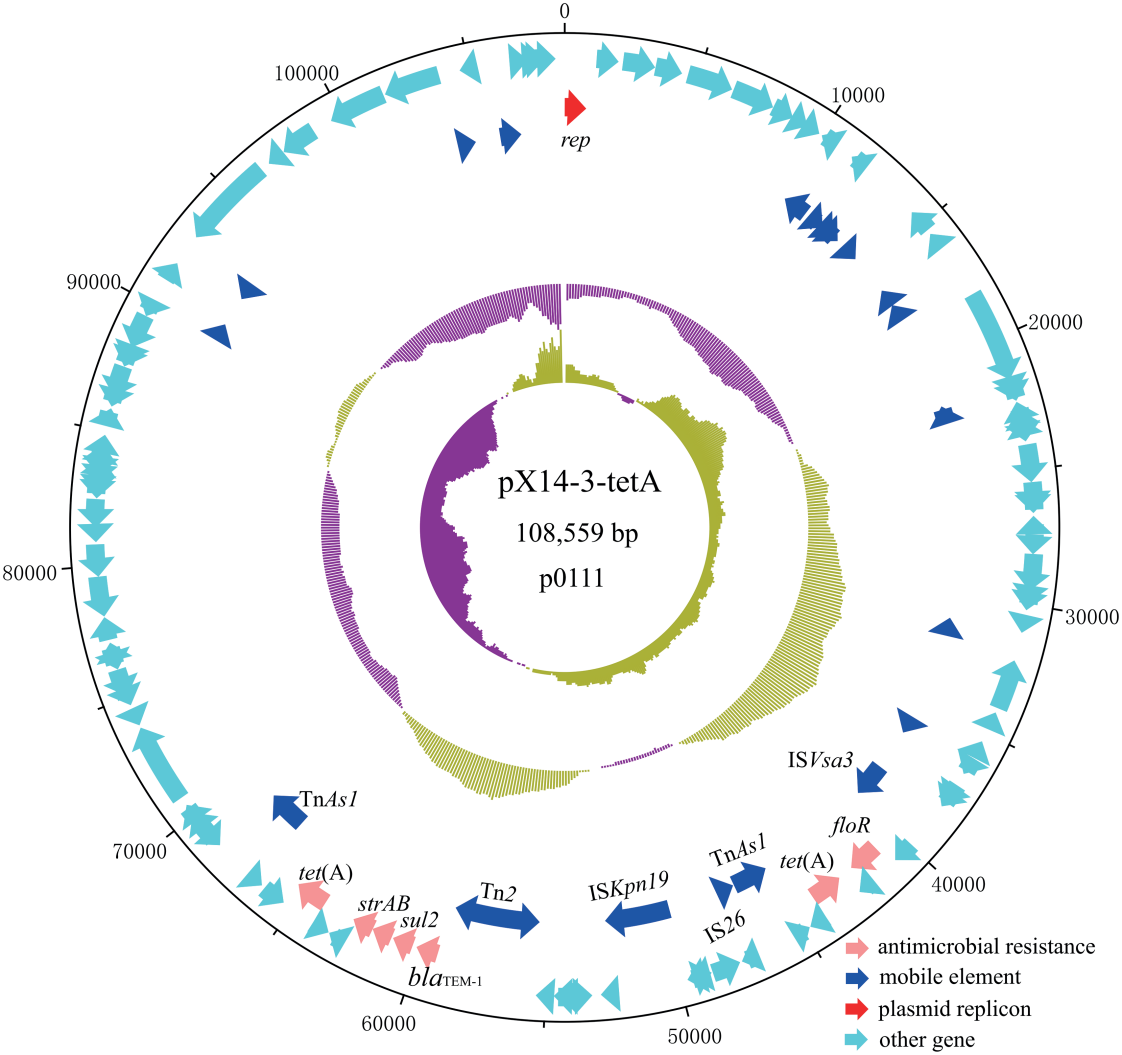
Circular plasmid map of pX14-3-tetA. Pink, blue, red, and cyan arrows represent genes responsible for antimicrobial resistance, mobile element, plasmid replicon, and other functions, respectively.

Plasmid pX14-3-tetA was a 108,559 bp, p0111 plasmid which encodes 123 predicted ORFs with a G + C content of 51.3%. It was 99.71% identical to the 192,477 bp plasmid p1079-IncFIB-N (accession: MG825383) from an *E. coli* isolate at 78% coverage. pX14-3-tetA carried antibiotic resistance genes including *strA*, *strB*, *sul2*, *bla*_TEM-1_, *floR,* and two copies of *tet*(A). These resistances genes were associated with diverse mobile elements including Tn*As1*, Tn*2*, IS*Kpn19*, IS*26,* and IS*Vsa3*, suggesting they were acquired by horizontal gene transfer (Figure 3).

## Discussion

Red swamp crayfish are crustaceans that not only play key roles in freshwater ecosystems but are also economically important (Dragičević et al., 2021). They are cultivated in aquaculture for both human consumption and ornamental purposes. Mounting evidence suggest that intestinal microbiota is closely correlated with host’s health status (Shui et al., 2020). Intestinal microbiota balance of crayfish could be affected by the external interferences, such as the mode of cultivation. A recent high-throughput 16S rRNA gene sequencing-based study suggested that development, followed by diet, is a better key driver for crayfish gut microbiota patterns than geographical location (Zhang et al., 2020; Xie et al., 2021). Also, healthy and diseased crayfish have distinct intestinal bacterial communities according to gut microbial community studies (Xiong et al., 2015). Sequence-based studies have demonstrated that the microflora of crayfish is high diverse, with dominant phyla including Proteobacteria, Bacteroidetes, Firmicutes, and Tenericutes (Shui et al., 2020; Zhang et al., 2020). Besides, Actinobacteria was reported to be dominant among crayfish sampled in rice fields but was not popular among those fed with fermented or non-fermented feed (Shui et al., 2020; Zhang et al., 2020). This could be related to the function of Actinobacteria which could breakdown plant biomass rich in rice fields (Zhang et al., 2020).

Compared with the sprouting studies on sequence-based research of crayfish intestinal bacteria, few studies reported bacteria in crayfish with culture-based methods. Culturable bacteria from crayfish are important resources for studying the function of intestinal microbiota. In this study, strains belonging to Gammaproteobacteria (*Citrobacter* sp., *Aeromonas* sp., *Pseudomonas* sp., *Morganella* sp., *Klebsiella* sp., *Acinetobacter* sp., *Proteus* sp., *Escherichia* sp., and *Kluyvera* sp.) and Bacteroidetes (*Myroides* sp.) were isolated from the intestines of healthy red swamp crayfish. According to previous studies, strains belonging to *Acinetobacter*, *Aeromonas*, *Citrobacter*, *Bacillus*, *Corynebacterium*, *Flavobacterium*, *Micrococcus*, *Pseudomonas*, *Staphylococcus,* and *Vibrio* could be isolated from crayfish (Lim et al., 2020; Liu et al., 2020; Feng et al., 2021; Wu et al., 2021). The bias between the species isolated in this study and previous ones could be associated with the isolation method including the culture media, temperature, and oxygen, and there is no ideal method for bacteria isolation (Tabacchioni et al., 2000). The high diversity of the gut microbiota of crayfish is a significant challenge for a culture dependent approach. We acknowledged the limitations in the isolation method in this study. More culture media and conditions could be tried to obtain more culturable bacteria from crayfish. *Citrobacter* sp. (36.6%) and *Aeromonas* sp. (34.2%) accounted for the majority of culturable bacteria from crayfish. *Citrobacter* sp. is a typical human-animal-fish comorbidity pathogen widely distributed in nature (Nawaz et al., 2008). *Aeromonas* sp. is a typical zoonosis and opportunistic pathogen widely distributed in the aquatic environment (Zhu et al., 2021). The increased breeding density and widespread use of antibiotics in aquaculture has led to the increasing prevalence of *Citrobacter* sp. and *Aeromonas* sp., which presents risks to crayfish farming (Patil et al., 2016; Liu et al., 2020).

Previous studies have sporadically reported the antimicrobial resistance of bacteria from diseased crayfish, but the antimicrobial resistance properties of culturable intestinal bacteria from healthy red swamp crayfish remained poorly studied (Ranjbar et al., 2019; Dong et al., 2020). Our results suggested most selected culturable bacteria from healthy crayfish were susceptible to all tested antibiotics, but a *Pseudomonas*. sp. an *A. caviae,* and an *E. coli* that were multidrug resistant were isolated. The farming mode which uses antibiotics in aquaculture systems could have posed pressure on intestinal bacteria and lead to the emergence of multidrug resistant strains. We further tested the presence of last-line antibiotic resistance genes among strains from crayfish intestine and found one *E. coli* belonging to ST48 with the carbapenemases gene, *bla*_NDM-5_. To our knowledge, this is the first report of *bla*_NDM-5_-positive *E. coli* isolate from red swamp crayfish. This isolate also carried an array of other antimicrobial resistance genes and a mercury resistance operon. *E. coli* with ST48 belonged to phylogroup A, and was frequently associated with antimicrobial resistance genes including last-line antibiotic resistance genes like carbapenemases genes and *mcr-1* (Wang et al., 2020). ST48 *E. coli* has been detected in healthy volunteers, seafood, and water (Aworh et al., 2021). Mobile genetic elements including plasmids, insertion sequences, integrons, and integrative and conjugative play pivotal roles in the dissemination of antimicrobial resistance (Partridge et al., 2018). In this study, *bla*_NDM-5_ was located on a IncHI1A/HI1B/FIA multi-replicon plasmid, constituting an intact Tn*3000*-like transposon with its adjacent genes. Tn*3000* belonged to the Tn*3* family which could actively mediate replicative transposition and have made major contributions to antimicrobial drug resistance dissemination or to endowing environmental bacteria with novel catabolic capacities (Nicolas et al., 2015). Previous study has demonstrated *bla*_NDM-1_ gene may be transmitted by Tn*3000* in different parts of the world (Campos et al., 2015). The presence of *bla*_NDM-5_ on such a mobile transposon poses a serious public health concern and suggests that new last-line antibiotic resistance genes are emerging in red swamp crayfish, which should be closely monitored.

## Data Availability Statement

The datasets presented in this study can be found in online repositories. The names of the repository/repositories and accession number(s) can be found at: https://www.ncbi.nlm.nih.gov/genbank/, CP084055, https://www.ncbi.nlm.nih.gov/genbank/, CP084057, and https://www.ncbi.nlm.nih.gov/genbank/, CP084056.

## Author Contributions

ZH and ND designed and conducted the experiments, prepared figures, and wrote the manuscript. YL contributed to review and edit the manuscript. CC contributed to development of methodology and validation. ND contributed to supervision, project administration, funding acquisition, and review and edit the manuscript. All authors contributed to the article and approved the submitted version.

## Funding

This work was supported by the start-up fund from Soochow University (GJ13400121).

## Conflict of Interest

The authors declare that the research was conducted in the absence of any commercial or financial relationships that could be construed as a potential conflict of interest.

## Publisher’s Note

All claims expressed in this article are solely those of the authors and do not necessarily represent those of their affiliated organizations, or those of the publisher, the editors and the reviewers. Any product that may be evaluated in this article, or claim that may be made by its manufacturer, is not guaranteed or endorsed by the publisher.

## References

[ref1] Aworh M. K. Kwaga J. K. Hendriksen R. S. Okolocha E. C. Thakur S. (2021). Genetic relatedness of multidrug resistant *Escherichia coli* isolated from humans, chickens and poultry environments. Antimicrob. Resist. Infect. Control 10, 1–13. doi: 10.1186/s13756-021-00930-x 33757589 PMC7988975

[ref2] Aziz R. K. Bartels D. Best A. A. Dejongh M. Disz T. Edwards R. A.. (2008). The RAST Server: rapid annotations using subsystems technology. BMC Genomics 9, 1–15. doi: 10.1186/1471-2164-9-75 18261238 PMC2265698

[ref3] Campos J. C. Da Silva M. J. F. Dos Santos P. R. N. Barros E. M. Pereira M. D. O. Seco B. M. S.. (2015). Characterization of Tn 3000, a transposon responsible for bla NDM-1 dissemination among Enterobacteriaceae in Brazil, Nepal, Morocco, and India. Antimicrob. Agents Chemother. 59, 7387–7395. doi: 10.1128/AAC.01458-15, PMID: 26392506 PMC4649174

[ref4] Carattoli A. Zankari E. García-Fernández A. Voldby Larsen M. Lund O. Villa L.. (2014). In silico detection and typing of plasmids using PlasmidFinder and plasmid multilocus sequence typing. Antimicrob. Agents Chemother. 58, 3895–3903. doi: 10.1128/AAC.02412-14, PMID: 24777092 PMC4068535

[ref5] Carver T. Thomson N. Bleasby A. Berriman M. Parkhill J. (2009). DNAPlotter: circular and linear interactive genome visualization. Bioinformatics 25, 119–120. doi: 10.1093/bioinformatics/btn578, PMID: 18990721 PMC2612626

[ref6] CLSI (2020). “Performance Standards for Antimicrobial Susceptibility Testing,” in CLSI Document M100. 30th ed (United States, PA: CLSI Wayne)

[ref7] Dong J. Zhang L. Zhou S. Xu N. Yang Q. Liu Y.. (2020). Identification of a multi-resistant Enterobacter cloacae strain from diseased crayfish (Procambarus clarkii). Aquacult. Rep. 17:100405. doi: 10.1016/j.aqrep.2020.100405

[ref8] Dragičević P. Bielen A. Petrić I. Hudina S. (2021). Microbial pathogens of freshwater crayfish: A critical review and systematization of the existing data with directions for future research. J. Fish Dis. 44, 221–247. doi: 10.1111/jfd.13314, PMID: 33345337

[ref9] Feng Y. Li M. Duan H. Li L. Ouyang P. Chen D.. (2021). Microbial analysis reveals the potential colonization of pathogens in the intestine of crayfish (Procambarus clarkii) in traditional aquaculture environments. Ecotoxicol. Environ. Saf. 224:112705. doi: 10.1016/j.ecoenv.2021.112705, PMID: 34454354

[ref10] He T. Wang R. Liu D. Walsh T. R. Zhang R. Lv Y.. (2019). Emergence of plasmid-mediated high-level tigecycline resistance genes in animals and humans. Nat. Microbiol. 4, 1450–1456. doi: 10.1038/s41564-019-0445-2, PMID: 31133751

[ref11] Hudzicki J. (2009). Kirby-Bauer disk diffusion susceptibility test protocol. Am. Soc. Microbiol. 15, 55–63.

[ref12] Jin S. Jacquin L. Xiong M. Li R. Lek S. Li W.. (2019). Reproductive pattern and population dynamics of commercial red swamp crayfish (Procambarus clarkii) from China: implications for sustainable aquaculture management. PeerJ 7:e6214. doi: 10.7717/peerj.6214, PMID: 30697477 PMC6347965

[ref13] Kleinheinz K. A. Joensen K. G. Larsen M. V. (2014). Applying the ResFinder and VirulenceFinder web-services for easy identification of acquired antibiotic resistance and E. coli virulence genes in bacteriophage and prophage nucleotide sequences. Bacteriophage 4:e27943. doi: 10.4161/bact.27943, PMID: 24575358 PMC3926868

[ref14] Li R. Peng K. Xiao X. Liu Y. Peng D. Wang Z. (2021). Emergence of a multidrug resistance efflux pump with carbapenem resistance gene bla VIM-2 in a Pseudomonas putida megaplasmid of migratory bird origin. J. Antimicrob. Chemother. 76, 1455–1458. doi: 10.1093/jac/dkab044, PMID: 33758948

[ref15] Li R. Xie M. Dong N. Lin D. Yang X. Wong M. H. Y.. (2018). Efficient generation of complete sequences of MDR-encoding plasmids by rapid assembly of MinION barcoding sequencing data. Gigascience 7:gix132 doi: 10.1093/gigascience/gix132 PMC5848804 29325009

[ref16] Lim Y. T. Yong A. S. K. Lim L. S. Al Azad S. Mohd Shah A. S. Lal T. M. (2020). Characterization and Identification of Bacteriocin-Like Substances Producing Lactic Acid Bacteria from the Intestine of Freshwater Crayfish, *Cherax quadricarinatus*. Int. J. Aquatic Science 11, 52–60.

[ref17] Liu X. He X. An Z. Sun W. Chen N. Gao X.. (2020). *Citrobacter freundii* infection in red swamp crayfish (Procambarus clarkii) and host immune-related gene expression profiles. Aquaculture 515:734499. doi: 10.1016/j.aquaculture.2019.734499

[ref18] Liu Y.-Y. Wang Y. Walsh T. R. Yi L.-X. Zhang R. Spencer J.. (2016). Emergence of plasmid-mediated colistin resistance mechanism MCR-1 in animals and human beings in China: a microbiological and molecular biological study. Lancet Infect. Dis. 16, 161–168. doi: 10.1016/S1473-3099(15)00424-7, PMID: 26603172

[ref19] Lv L. Wan M. Wang C. Gao X. Yang Q. Partridge S. R.. (2020). Emergence of a plasmid-encoded resistance-nodulation-division efflux pump conferring resistance to multiple drugs, including tigecycline, in Klebsiella pneumoniae. mBio 11, e02930–e02919. doi: 10.1128/mBio.02930-19, PMID: 32127452 PMC7064769

[ref20] Ministry-of-Agriculture-and-Rural-Affairs-of-The-People’s-Republic-of-China (2021). China Crayfish Industry Development Report [Online]. Available at: http://www.moa.gov.cn/ (Accessed September 1, 2021).

[ref21] Nawaz M. Khan A. A. Khan S. Sung K. Steele R. (2008). Isolation and characterization of tetracycline-resistant Citrobacter spp. from catfish. Food Microbiol. 25, 85–91. doi: 10.1016/j.fm.2007.07.008, PMID: 17993380

[ref22] Nicolas E. Lambin M. Dandoy D. Galloy C. Nguyen N. Oger C. A.. (2015). The Tn 3-family of replicative transposons. Microbiol. Spectrum 3:2014. doi: 10.1128/microbiolspec.MDNA3-0060-2014, PMID: 26350313

[ref23] Partridge S. R. Kwong S. M. Firth N. Jensen S. O. (2018). Mobile genetic elements associated with antimicrobial resistance. Clin. Microbiol. Rev. 31, e00088–e00017. doi: 10.1128/CMR.00088-17 30068738 PMC6148190

[ref24] Patil H. J. Benet-Perelberg A. Naor A. Smirnov M. Ofek T. Nasser A.. (2016). Evidence of increased antibiotic resistance in phylogenetically-diverse Aeromonas isolates from semi-intensive fish ponds treated with antibiotics. Front. Microbiol. 7:1875 doi: 10.3389/fmicb.2016.01875 27965628 PMC5124577

[ref25] Poirel L. Walsh T. R. Cuvillier V. Nordmann P. (2011). Multiplex PCR for detection of acquired carbapenemase genes. Diagn. Microbiol. Infect. Dis. 70, 119–123. doi: 10.1016/j.diagmicrobio.2010.12.002, PMID: 21398074

[ref26] Ranjbar R. Salighehzadeh R. Sharifiyazdi H. (2019). Antimicrobial resistance and incidence of integrons in Aeromonas Species isolated from diseased freshwater animals and water samples in Iran. Antibiotics 8:198. doi: 10.3390/antibiotics8040198, PMID: 31661794 PMC6963716

[ref27] Seemann T. (2021). mlst [Online]. Available at: https://github.com/tseemann/mlst (Accessed September 1, 2021).

[ref28] Shui Y. Guan Z.-B. Liu G.-F. Fan L.-M. (2020). Gut microbiota of red swamp crayfish Procambarus clarkii in integrated crayfish-rice cultivation model. AMB Express 10, 1–11. doi: 10.1186/s13568-019-0944-9 PMC6960274 31938890

[ref29] Siguier P. Pérochon J. Lestrade L. Mahillon J. Chandler M. (2006). ISfinder: the reference centre for bacterial insertion sequences. Nucleic Acids Res. 34, D32–D36. doi: 10.1093/nar/gkj014, PMID: 16381877 PMC1347377

[ref30] Singh A. K. Das S. Kumar S. Gajamer V. R. Najar I. N. Lepcha Y. D.. (2020). Distribution of Antibiotic-Resistant Enterobacteriaceae Pathogens in Potable Spring Water of Eastern Indian Himalayas: Emphasis on Virulence Gene and Antibiotic Resistance Genes in *Escherichia coli*. Front. Microbiol. 11:581072. doi: 10.3389/fmicb.2020.581072 33224119 PMC7674312

[ref31] Sony M. Sumithra T. Anusree V. Amala P. Reshma K. Alex S.. (2021). Antimicrobial resistance and virulence characteristics of Vibrio vulnificus, Vibrio parahaemolyticus and Vibrio harveyi from natural disease outbreaks of marine/estuarine fishes. Aquaculture 539:736608. doi: 10.1016/j.aquaculture.2021.736608

[ref32] Sullivan M. J. Petty N. K. Beatson S. A. (2011). Easyfig: a genome comparison visualizer. Bioinformatics 27, 1009–1010. doi: 10.1093/bioinformatics/btr039, PMID: 21278367 PMC3065679

[ref33] Sun J. Chen C. Cui C.-Y. Zhang Y. Liu X. Cui Z.-H.. (2019). Plasmid-encoded tet (X) genes that confer high-level tigecycline resistance in *Escherichia coli*. Nat. Microbiol. 4, 1457–1464. doi: 10.1038/s41564-019-0496-4, PMID: 31235960 PMC6707864

[ref34] Sun S. Korheina D. K. Fu H. Ge X. (2020). Chronic exposure to dietary antibiotics affects intestinal health and antibiotic resistance gene abundance in oriental river prawn (Macrobrachium nipponense), and provokes human health risk. Sci. Total Environ. 720:137478. doi: 10.1016/j.scitotenv.2020.137478, PMID: 32145616

[ref35] Tabacchioni S. Chiarini L. Bevivino A. Cantale C. Dalmastri C. (2000). Bias caused by using different isolation media for assessing the genetic diversity of a natural microbial population. Microb. Ecol. 40, 169–176. doi: 10.1007/s002480000015, PMID: 11080375

[ref36] Tan Y. Peng B. Wu Y. Xiong L. Sun J. Peng G.. (2021). Human health risk assessment of toxic heavy metal and metalloid intake via consumption of red swamp crayfish (Procambarus clarkii) from rice-crayfish co-culture fields in China. Food Control 128:108181. doi: 10.1016/j.foodcont.2021.108181

[ref37] Wang Q. Ding H. Tao Z. Ma D. (2018). Crayfish (Procambarus clarkii) cultivation in China: a decade of unprecedented development. Aquacult. China, 363–377. doi: 10.1002/9781119120759.ch4_1

[ref38] Wang Y. Liu H. Wang Q. Du X. Yu Y. Jiang Y. (2020). Coexistence of blaKPC-2–IncN and mcr-1–IncX4 plasmids in a ST48 *Escherichia coli* strain in China. J. Global Antimicrob. Resis. 23, 149–153. doi: 10.1016/j.jgar.2020.08.023, PMID: 32966910

[ref39] Wick R. R. Judd L. M. Gorrie C. L. Holt K. E. (2017). Unicycler: resolving bacterial genome assemblies from short and long sequencing reads. PLoS Comput. Biol. 13:e1005595. doi: 10.1371/journal.pcbi.1005595, PMID: 28594827 PMC5481147

[ref40] Wu Z. Zhang Q. Zhang T. Chen J. Wang S. Hao J.. (2021). Association of the microbiota dysbiosis in the hepatopancreas of farmed crayfish (Procambarus clarkii) with disease outbreaks. Aquaculture 536:736492. doi: 10.1016/j.aquaculture.2021.736492

[ref41] Xie M. Zhang S. Xu L. Wu Z. Yuan J. Chen X. (2021). Comparison of the intestinal microbiota during the different growth stages of red swamp crayfish (Procambarus clarkii). Front. Microbiol. 12:696281. doi: 10.3389/fmicb.2021.696281, PMID: 34589066 PMC8473915

[ref42] Xiong J. Wang K. Wu J. Qiuqian L. Yang K. Qian Y.. (2015). Changes in intestinal bacterial communities are closely associated with shrimp disease severity. Appl. Microbiol. Biotechnol. 99, 6911–6919. doi: 10.1007/s00253-015-6632-z, PMID: 25947250

[ref43] Zhang R. Dong N. Huang Y. Zhou H. Xie M. Chan E. W.-C.. (2018). Evolution of tigecycline-and colistin-resistant CRKP (carbapenem-resistant Klebsiella pneumoniae) in vivo and its persistence in the GI tract. Emerg. Microb. Infect. 7, 1–11. doi: 10.1038/s41426-018-0129-7 PMC6037711 29985412

[ref44] Zhang Z. Liu J. Jin X. Liu C. Fan C. Guo L.. (2020). Developmental, dietary, and geographical impacts on gut microbiota of red swamp crayfish (Procambarus clarkii). Microorganisms 8:1376. doi: 10.3390/microorganisms8091376, PMID: 32911609 PMC7565139

[ref45] Zhu L. Wang X. Hou L. Jiang X. Li C. Zhang J.. (2021). The related immunity responses of red swamp crayfish (Procambarus clarkii) following infection with *Aeromonas veronii*. Aquacult. Rep. 21:100849. doi: 10.1016/j.aqrep.2021.100849

