# Supplementary material for: Isolation, Molecular Characterization, and Antimicrobial Resistance of Selected Culturable Bacteria From Crayfish (Procambarus clarkii)
Source: Front Microbiol. 2022 Jun 7;13:911777. doi: 10.3389/fmicb.2022.911777 (PMC9209738; doi:10.3389/fmicb.2022.911777)
Supplement: Supplementary file 1 [file Data_Sheet_1.pdf]

Supplementary Table 1. Primers used for screening of antibiotic resistance genes

Primer	Sequence (5′–3′)	Gene	Product size (bp)	Reference
VIM-F	GATGGTGTTTGGTCGCATA	$\textit{bla}_{\text{VIM}}$	390	[1]
VIM-R	CGAATGCGCAGCACCAG			
NDM-F	GGTTTGGCGATCTGGTTTTC	$\textit{bla}_{\text{NDM}}$	699	[1]
NDM-R	CGGAATGGCTCATCACGATC			
IMP-F	GGAATAGAGTGGCTTAAYTCTC	$\textit{bla}_{\text{IMP}}$	232	[1]
IMP-R	GGTTTAAYAAAACAACCACC			
OXA-F	GCGTGGTTAAGGATGAACAC	$\textit{bla}_{\text{OXA-48}}$	438	[1]
OXA-R	CATCAAGTTCAACCCAACCG			
KPC-F	ATGTCACTGTATCGCCGTCT	$\textit{bla}_{\text{KPC}}$	894	[2]
KPC-R	TTTTCAGAGCCTTACTGCCC			
mcr1-F	AGTCCGTTTGTTCTTGTGGC	*mcr-1*	320	[3]
mcr1-R	AGATCCTTGGTCTCGGCTTG			
tetX-F	TTAGCCTTACCAATGGGTGT	*tet*(X)	243	[4]
tetX-R	CAAATCTGCTGTTTCACTCG			
tmexD-F	CTGCTGGTCATTCCGTTCCT	*tmexCD1-*	1196	[5]
tmexD-R	ATGATCCGCTCGACGTTCTC	*toprJ1*		

References

[1] Poirel, L., Walsh, T.R., Cuvillier, V., Nordmann, P., 2011. Multiplex PCR for detection of acquired carbapenemase genes. Diagn. Microbiol. Infect. Dis. 70, 119-123.

[2] Schechner, V., Straus-Robinson, K., Schwartz, D., et al., 2009. Evaluation of PCR-based testing for surveillance of KPC-producing carbapenem-resistant members of the Enterobacteriaceae family. J. Clin. Microbiol. 47(10), 3261-3265.

[3] Liu, Y.-Y., Wang, Y., Walsh, T.R., Yi, L.-X., Zhang, R., Spencer, J., Doi, Y., Tian, G., Dong, B., Huang, X., 2016. Emergence of plasmid-mediated colistin resistance mechanism MCR-1 in animals and human beings in China: a microbiological and molecular biological study. The Lancet Infect. Dis. 16, 161-168.

[4] Bartha, N.A., Sóki, J., Urbán, E., et al., 2011. Investigation of the prevalence of tetQ, tetX and tetX1 genes in Bacteroides strains with elevated tigecycline minimum inhibitory concentrations. Int. J. Antimicrob. Agents., 38(6), 522-525.

[5] Li, R., Peng, K., Xiao, X., Liu, Y., Peng, D., Wang, Z., 2021. Emergence of a multidrug resistance efflux pump with carbapenem resistance gene bla VIM-2 in a Pseudomonas putida megaplasmid of migratory bird origin. J. Antimicrob. Chemother. 76, 1455-1458.

Supplementary Table 2. Bacterial species and numbers isolated from each red swamp crayfish

Crayfish ID	isolate 1	isolate 2	isolate 3	isolate 4	isolate 5	isolate 6	Number of isolates
1	*Citrobacter braakii*	*Aeromonas enteropelogenes*	*Pseudomonas putida*				3
2	*Citrobacter braakii*	*Aeromonas enteropelogenes*	*Aeromonas jandaei*				3
3	*Citrobacter braakii*	*Aeromonas enteropelogenes*					2
4	*Citrobacter braakii*	*Aeromonas enteropelogenes*	*Pseudomonas putida*				3
5	*Citrobacter braakii*	*Aeromonas enteropelogenes*	*Aeromonas hydrophila*				3
6	*Citrobacter braakii*						1
7	*Citrobacter braakii*	*Klebsiella pneumoniae*	*Aeromonas veronii*	*Myroides odoratimimus*	*Morganella morganii*	*Pseudomonas putida*	6
8	*Citrobacter braakii*	*Pseudomonas sp.*					2
9	*Citrobacter braakii*	*Acinetobacter radioresistens*					2
10	*Citrobacter braakii*	*Aeromonas hydrophila*					2
11	*Citrobacter braakii*	*Aeromonas caviae*	*Morganella morganii*	*Acinetobacter lwoffii*			4
12	*Citrobacter braakii*	*Aeromonas caviae*					2
13	*Citrobacter braakii*	*Aeromonas caviae*					2
14	*Citrobacter braakii*	*Aeromonas caviae*	*Pseudomonas sp.*				3
15	*Citrobacter braakii*	*Aeromonas caviae*	*Aeromonas hydrophila*				3
16	*Citrobacter braakii*	*Aeromonas veronii*	*Myroides odoratimimus*	*Pseudomonas mosselii*	*Pseudomonas putida*	*Escherichia coli*	6
17	*Citrobacter braakii*	*Aeromonas veronii*					2
18	*Citrobacter braakii*	*Aeromonas veronii*					2
19	*Citrobacter braakii*	*Aeromonas veronii*					2
20	*Citrobacter braakii*	*Aeromonas veronii*					2
21	*Citrobacter braakii*	*Aeromonas veronii*					2
22	*Citrobacter braakii*						1

23	*Aeromonas enteropelogenes*						1
24	*Aeromonas enteropelogenes*						1
25	*Pseudomonas putida*						1
26	*Klebsiella pneumoniae*	*Aeromonas enteropelogenes*	*Pseudomonas putida*				3
27	*Klebsiella pneumoniae*	*Aeromonas veronii*	*Aeromonas hydrophila*				3
28	*Klebsiella pneumoniae*	*Aeromonas veronii*	*Aeromonas hydrophila*				3
29	*Aeromonas caviae*						1
30	*Citrobacter fraudii*	*Acinetobacter sp.*					2
31	*Citrobacter fraudii*	*Acinetobacter sp.*					2
32	*Citrobacter fraudii*	*Acinetobacter sp.*					2
33	*Citrobacter fraudii*	*Citrobacter braakii*					2
34	*Citrobacter fraudii*	*Citrobacter braakii*	*Klebsiella pneumoniae*	*Morganella morganii*			4
35	*Citrobacter fraudii*	*Citrobacter braakii*	*Morganella morganii*				3
36	*Citrobacter fraudii*	*Citrobacter braakii*	*Aeromonas caviae*	*Pseudomonas putida*	*Proteus vulgaris*	*Proteus hauseri*	6
37	*Citrobacter fraudii*	*Citrobacter braakii*	*Aeromonas veronii*	*Myroides odoratimimus*	*Morganella morganii*	*Citrobacter youngae*	6
38	*Citrobacter fraudii*	*Citrobacter braakii*	*Klebsiella pneumoniae*	*Aeromonas veronii*	*Aeromonas enteropelogenes*	*Myroides odoratimimus*	6
39	*Citrobacter fraudii*	*Citrobacter braakii*	*Aeromonas veronii*	*Morganella morganii*			4
40	*Citrobacter fraudii*	*Citrobacter braakii*					2
41	*Citrobacter fraudii*	*Citrobacter braakii*					2
42	*Citrobacter fraudii*	*Aeromonas enteropelogenes*	*Aeromonas jandaei*	*Myroides odoratimimus*			4
43	*Citrobacter fraudii*	*Aeromonas enteropelogenes*					2
44	*Citrobacter fraudii*	*Aeromonas enteropelogenes*					2
45	*Citrobacter fraudii*						1
46	*Citrobacter fraudii*	*Pseudomonas putida*					2
47	*Citrobacter fraudii*	*Pseudomonas putida*					2
48	*Citrobacter fraudii*	*Pseudomonas putida*					2

49	*Citrobacter fraudii*	*Pseudomonas putida*					2
50	*Citrobacter fraudii*	*Pseudomonas putida*					2
51	*Citrobacter fraudii*	*Pseudomonas putida*	*Klebsiella aerogenes*				3
52	*Citrobacter fraudii*	*Klebsiella pneumoniae*	*Aeromonas veronii*	*Morganella morganii*			4
53	*Citrobacter fraudii*	*Pseudomonas sp.*					2
54	*Citrobacter fraudii*	*Pseudomonas sp.*					2
55	*Citrobacter fraudii*	*Pseudomonas sp.*					2
56	*Citrobacter fraudii*	*Aeromonas jandaei*					2
57	*Citrobacter fraudii*	*Morganella morganii*					2
58	*Citrobacter fraudii*	*Myroides odoratimimus*					2
59	*Citrobacter fraudii*	*Myroides odoratimimus*	*Morganella morganii*	*Citrobacter youngae*			4
60	*Citrobacter fraudii*	*Myroides odoratimimus*					2
61	*Citrobacter fraudii*	*Myroides odoratimimus*					2
62	*Citrobacter fraudii*	*Kluyvera cryocrescens*					2
63	*Citrobacter fraudii*	*Aeromonas hydrophila*					2
64	*Citrobacter fraudii*	*Pseudomonas monteilii*					2
65	*Citrobacter fraudii*	*Aeromonas caviae*					2
66	*Citrobacter fraudii*	*Aeromonas caviae*	*Escherichia coli*				3
67	*Citrobacter fraudii*	*Aeromonas caviae*	*Aeromonas hydrophila*				3
68	*Citrobacter fraudii*	*Aeromonas veronii*	*Aeromonas enteropelogenes*	*Myroides odoratimimus*	*Acinetobacter baumannii*	*Pseudomonas mosselii*	6
69	*Citrobacter fraudii*	*Aeromonas veronii*	*Aeromonas caviae*				3
70	*Citrobacter fraudii*	*Aeromonas veronii*	*Aeromonas hydrophila*	*Morganella morganii*			4
71	*Citrobacter fraudii*	*Aeromonas veronii*	*Pseudomonas putida*	*Pseudomonas monteilii*	*Proteus mirabilis*		5
72	*Citrobacter fraudii*	*Aeromonas veronii*	*Myroides odoratimimus*				3
73	*Citrobacter fraudii*	*Aeromonas veronii*	*Aeromonas enteropelogenes*				3
74	*Citrobacter fraudii*	*Aeromonas veronii*					2

75	*Citrobacter fraudii*	*Aeromonas veronii*			2
76	*Citrobacter fraudii*	*Aeromonas veronii*			2
77	*Citrobacter fraudii*	*Aeromonas veronii*			2
78	*Citrobacter fraudii*	*Aeromonas veronii*	*Aeromonas jandaei*		3
79	*Citrobacter fraudii*	*Aeromonas veronii*	*Pseudomonas sp.*		3
80	*Citrobacter fraudii*				1
81	*Citrobacter fraudii*				1
82	*Citrobacter fraudii*				1
83	*Citrobacter fraudii*				1
84	*Citrobacter fraudii*				1
85	*Citrobacter fraudii*				1
86	*Citrobacter fraudii*				1
87	*Citrobacter fraudii*				1
88	*Citrobacter fraudii*				1
89	*Citrobacter fraudii*				1
90	*Citrobacter braakii*				1
91	*Aeromonas jandaei*				1
92	*Aeromonas jandaei*	*Proteus vulgaris*			2
93	*Myroides odoratimimus*	*Pseudomonas putida*	*Kluyvera georgiana*	*Pseudomonas otitidis*	4
94	*Aeromonas hydrophila*				1
95	*Aeromonas caviae*				1
96	*Aeromonas caviae*	*Aeromonas hydrophila*			2
97	*Aeromonas caviae*				1
98	*Aeromonas caviae*				1
99	*Aeromonas caviae*				1
100	*Aeromonas veronii*	*Aeromonas enteropelogenes*			2

101	*Aeromonas veronii*	*Pseudomonas putida*		2
102	*Aeromonas veronii*	*Pseudomonas monteilii*		2
103	*Aeromonas veronii*	*Aeromonas hydrophila*	*Pseudomonas putida*	3
104	*Aeromonas veronii*	*Myroides odoratimimus*		2
105	*Aeromonas veronii*			1
106	*Aeromonas veronii*			1
107	*Aeromonas veronii*			1
108	*Aeromonas veronii*			1
109	*Aeromonas veronii*			1
110	*Aeromonas veronii*			1
111	*Aeromonas veronii*			1
112	*Aeromonas veronii*			1
113	*Aeromonas veronii*			1
114	*Aeromonas veronii*			1
115	*Escherichia coli*			1
